# Clinical and Radiological Results after Fracture-Dislocations of the Ankle: A Medium- to Long-Term Followup Study

**DOI:** 10.3390/jfmk7020030

**Published:** 2022-03-25

**Authors:** Vincenzo De Luna, Alessandro Caterini, Chiara Casci, Martina Marsiolo, Kristian Efremov, Fernando De Maio, Pasquale Farsetti

**Affiliations:** Department of Clinical Science and Translational Medicine, Section of Orthopaedics and Traumatology, University of Rome “Tor Vergata”, 00133 Rome, Italy; videluna@hotmail.com (V.D.L.); caterini.alessandro@gmail.com (A.C.); cascichiara@gmail.com (C.C.); marsiolom@gmail.com (M.M.); efremovk@yahoo.com (K.E.); demaio@med.uniroma2.it (F.D.M.)

**Keywords:** fracture, unimalleolar, bimalleolar, trimalleolar, dislocation, ankle

## Abstract

The authors report the long-term outcome in a series of 26 patients surgically treated for a fracture-dislocation of the ankle by open reduction and internal fixation (ORIF), reviewed after an average followup of 5.2 years. The average age of the patients was 46.8 years; 17 were female and 9 male; the right side was involved in 22 patients and the left side in 4; 10 patients had a unimalleolar fracture, 11 a bimalleolar fracture, and 5 a trimalleolar fracture. The quality of reduction was excellent in 14 cases and good in 12. The functional results were assessed using the American Orthopaedic Foot and Ankle Society (AOFAS) score, while radiographic results followed the Van Dijk classification. At followup, the AOFAS score ranged from 75 to 98 points with an average of 87.9, while the radiographic results were evaluated as grade 0 in 16 ankles and grade I in 10. Fracture-dislocations of the ankle occurred more frequently after high-energy traumas in younger patients on the right side, but they were also observed in older females after low-energy trauma. Excellent reduction was correlated with better radiographic results at long term followup. However, these injuries may lead to a poor functional outcome, despite an anatomical reduction and good radiographic results; in fact, in nine of our cases (34.6 percent), the AOFAS score was less than 90 points.

## 1. Introduction

Ankle fractures are one of the most common injuries of the lower limb and usually affect young men and old women. This lesion represents 10% of all traumatic fractures [1,2,3,4,5,6,7]. Ankle fractures may occur with and without dislocation; however, fracture-dislocation of the ankle represents an uncommon lesion caused by severe trauma, which involves bony and soft tissues around the ankle. Immediate closed reduction of the ankle dislocation and temporary immobilization with an external fixator or bivalved cast, to maintain stabilization postreduction, is a crucial step in the initial management of these severe injuries while awaiting definitive synthesis. This initial procedure plays an important role in minimizing soft tissue and neurovascular complications. Open reduction and internal fixation is the first choice for treatment of these severe injuries. The final goal of surgical treatment is to restore normal anatomy with the aim to minimize joint stiffness, residual pain, and posttraumatic osteoarthritis, which represent the major complications of these lesions. However, surgical restoration of the normal anatomy may still be associated with unsatisfactory results. Other factors may predispose to poor clinical and radiographic results such as comorbidities, age, sex, and energy level of the trauma. Fracture classification of the ankle is an important predictor of clinical and radiographic outcome after surgery. The Danis-Weber classification system represents one of the most widely adopted classification systems, classifying an ankle fracture into one of three types (A, B, and C), according to the location of the lateral malleolus fracture. In type A, the fracture of the lateral malleolus is under the syndesmosis, in type B, it is trans-syndesmotic, and in type C, it is above the syndesmosis. Type A and B fractures are stable, while type C is unstable and may be fixed with trans-syndesmotic screws [8,9,10,11,12]. Few studies have reported the medium- or long-term outcomes of surgically-treated fracture-dislocations of the ankle [11,12]. The aim of our study was to report the long-term results in 26 patients affected by a fracture-dislocation of the ankle surgically treated by open reduction and internal fixation, with a medium followup time of 5.2 years.

## 2. Materials and Methods

**Figure 1:**
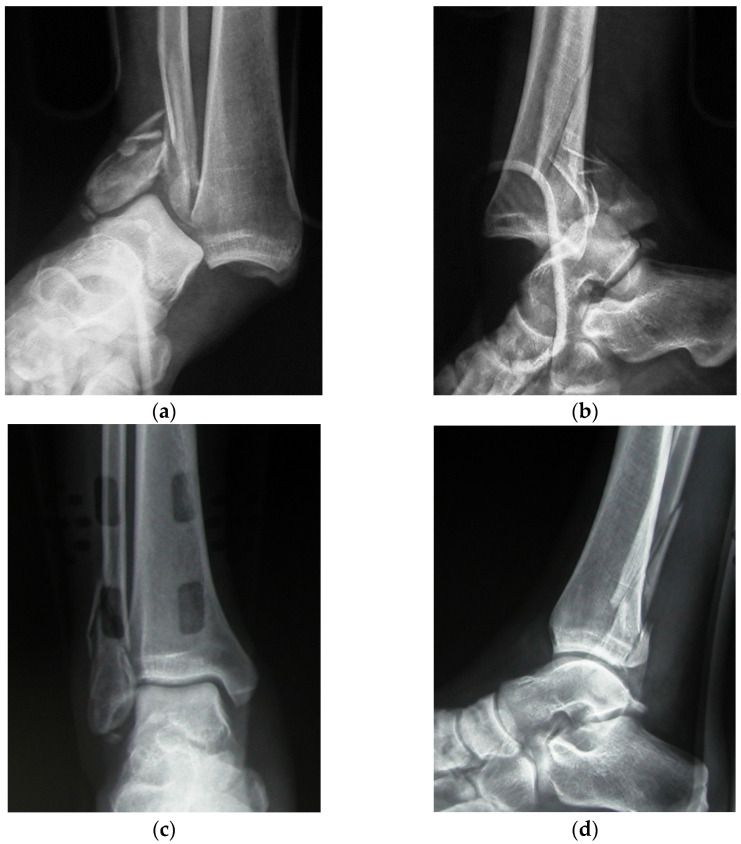
Radiographic examination of a fracture-dislocation of the right ankle in a 32 year-old man (**a**,**b**), reduced in the emergency room and temporarily stabilized by a bivalved cast (**c**,**d**).

From a pool of 107 ankle fractures treated between January 2009 and December 2015 at our institution, we identified a total of 26 patients affected by a fracture-dislocation of the ankle. Open fractures were excluded. Nine patients were males, and 17 were females, with an average age of 46.8 years (range 16 to 79 years). At admission in the emergency room, a radiographic examination of the ankle in three views was performed in all patients, while 11 patients additionally underwent a CT scan because the fracture was more complex, and standard radiographic examination was not sufficient to make an exact diagnosis. A fracture-dislocation was diagnosed when the radiographs or CT scan showed no joint apposition of the tibia and the talus, which were completely dislocated. The ankle dislocation was associated with a unimalleolar fracture in 10 cases, a bimalleolar fracture in 11 cases, and a trimalleolar in 5. We define “unimalleolar” as a fracture of medial or lateral malleolus; “bimalleolar” as a fracture of the medial and lateral malleolus, and “trimalleolar” as a fracture of the medial, lateral, and posterior malleolus. No Bosworth fracture-dislocation was observed [13], in which a bimalleolar fracture-dislocation of the ankle is associated with entrapment of the proximal fibular fragment behind the posterior malleolus. Closed reduction of the dislocation was performed in all cases in the emergency room under general anesthesia. A temporary cast immobilization below the knee was applied for maintaining the reduction obtained while waiting for definitive osteosynthesis (Figure 1). According the Danis-Weber classification, one case was Weber A, 15 cases were Weber B, and 10 were Weber C. Operative treatment by open reduction and internal fixation (ORIF) was performed in all cases after a mean time of 2.4 days. Fractures of the fibula were reduced and stabilized first by a plate; then, fractures of the medial and posterior malleolus were stabilized with cannulated screws. Syndesmotic instability, when present, was treated by trans-syndesmotic screws that were removed after 4–6 weeks. We used anatomical shaped plates, with 3.5 or 2.8 mm screws for fibular fractures and 4 mm cannulated screws for medial and posterior malleoli. We reduced medial malleolus fractures through an open access, while posterior malleolus fractures were reduced percutaneously. We used tricortical 3.5 screws to fix the syndesmosis. Lesions of the peritalar ligament complex were sutured with resorbable sutures. All patients received prophylactic antibiotic therapy. After surgery, a postoperative ankle radiograph was performed in all patients to assess the quality of the reduction; then, the ankle was immobilized in a plantar splint below the knee for 3 weeks. The quality of the reduction was assessed by measuring the medial clear space between the lateral border of the medial malleolus and the medial border of the talus. It was considered excellent when the articular space was less than 2 mm, good when it was between 2 and 3 mm, and unsatisfactory when it measured more than 3 mm. Subsequently, the patients started physical rehabilitation of the ankle and progressive weight bearing was permitted after 6 weeks. Clinical and radiological followup were performed after 4, 8, and 12 weeks after surgery. Some patients who needed further checks were followed up in our hospital, even after 12 weeks.

Fracture healing was assessed by clinical and radiographic examination criteria. Clinically, we evaluated presence or absence of pain at the fracture site on weight bearing, while radiographically, presence or absence of callus and fracture line.

At the last followup, performed in our hospital, clinical results were assessed using the American Orthopaedic Foot and Ankle Society Score (AOFAS) which analyzes range of motion (ROM), stability of the ankle, pain, other symptoms, activities of daily living, sport, and quality of life [14]. Posttraumatic osteoarthritis was evaluated by radiographic examination of the ankle using the Van Dijk criteria (grade 0: normal joint or subchondral sclerosis; grade I: presence of osteophytes without joint space narrowing; grade II: joint space narrowing with or without osteophytes; grade III: (sub) total disappearance or deformation of the joint space [15].

### Statistical Analysis

Statistical analysis was performed to find any correlations between the initial data, which included age, sex, the energy mechanism, the type of fracture, the Webber classification, and the quality of the reduction and the followup results, consisting of the AOFAS and the Van Dijk criteria. The Student’s *t*-test and Mann-Whitney U test were used to evaluate the significance of differences for continuous variables (AOFAS). A chi-squared test or Fisher’s exact test was used to evaluate the significance of differences of the categorical variables (Van Dijk criteria). All statistical analyses were performed using the SigmaStat Version 4.0 Program (Systat Software, San Jose, CA, USA); *p*-values less than 0.05 were considered significant.

## 3. Results

In Table 1 we report all our cases with the type of trauma, the type of fracture, the quality of the reduction and the clinical and radiographic result.

Fracture healing was obtained in all cases after an average time of 9 weeks after surgery (range 6–12 weeks). Major wound complications occurred in 2 of our patients that healed after antibiotic therapy and hardware removal, performed at least 12 weeks after surgery.

**Figure 2:**
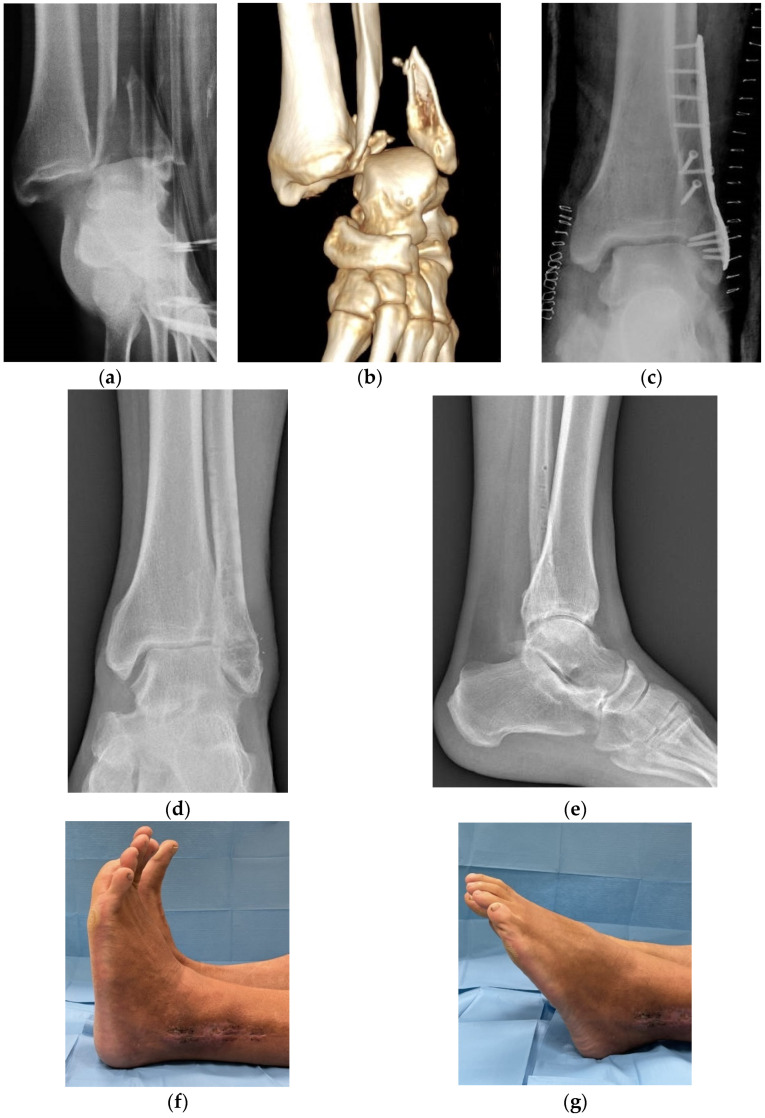
Radiographic examination with a 3D CT scan reconstruction of **a** fracture-dislocation of the left ankle in a 45 year-old man (**a**,**b**) treated by open reduction and internal fixation. The post-operative X-rays showed an anatomic reduction of the lesion with a stable osteosynthesis (**c**). At follow up, performed 3.8 years later, after hardware removal, AOFAS was 85 points, in spite of a good range of motion of the ankle and the absence of significant radiographic signs of osteoarthritis (Van Dick grade 0) (**d**–**g**).

At final followup, after a mean of 5.2 years, the AOFAS scores ranged from 90 to 98 in 11 patients (Figure 2a–g), from 80 to 88 in 13 patients, and below 80 in 2 patients. At radiographic examination, no significant signs of osteoarthritis were observed in 16 patients, whereas moderate osteoarthritis was present in the remaining 10. 

There was a statistically significant correlation between age and energy mechanism, with the low energy fractures having an average age of 73.8 years, while the high energy averaged 40.3 years (*p* < 0.001). No other correlations were observed between the initial characteristics.

The AOFAS was not observed to be statistically correlated with the age or sex of the patient, amount of energy (low or high), the type of fracture (uni, bi or trimalleolar), the Weber Classification (A, B or C), or the quality of reduction (good or excellent). 

The radiographic results via the Van Dijk criteria were significantly correlated with the quality of reduction, with the better reduced fracture-dislocations exhibiting lower arthritis scores (*p* > 0.001). Low energy fractures were significantly correlated with higher osteoarthritis scores (*p* = 0.01). No other initial data were significantly associated to the radiographic results.

In addition, the AOFAS was not significantly correlated with the radiographic OA results.

## 4. Discussion

Few studies have reported medium- or long-term results in fracture-dislocation of the ankle [9,11,12,16]. Warner et al. [9] compared short-term functional outcomes in pronation external rotation ankle fractures with and without dislocation. Of the 47 fractures included in the study, 20 (43%) were fracture-dislocations and 27 (57%) had no dislocation. The mean age of the study cohort was 49 years (range 24–91 years), and all fractures were surgically treated. At followup, the fracture-dislocation cohort demonstrated significantly poorer functional outcomes than the non-dislocation cohort, regarding symptoms, pain, activities of daily living, sports, and quality of life. The authors concluded that dislocation generally occurred after a high energy injury and the articular cartilage damage which occurred led to the unfavorable prognosis of these injuries. Sculco et al. [11], in a retrospective study of 108 patients surgically treated for a supination external rotation type of ankle fracture, evaluated the effect of dislocation on postoperative outcomes. Fractures were divided into two groups based on clinical and radiographic evidence of dislocation; 73 patients (68%) had no dislocation, while 35 (32%) were associated with a dislocation. At a mean followup of 21 months, ankle fracture-dislocation patients had increased pain and decreased activity of daily living based on the FAOS score (Foot and Ankle Outcome Score) and significantly worse ankle and subtalar ROM. The authors concluded that concurrent dislocation at the time of ankle fracture was associated with worse outcomes. All our patients had a fracture-dislocation of the ankle, in some cases caused by low energy trauma, especially in elderly patients. Tantigate et al. [12] reported a retrospective study of 118 patients surgically treated for an ankle fracture; 33 patients (28%) sustained a fracture-dislocation. After a medium followup of 37 months, 62 patients were analyzed for functional outcome assessed by the Foot and Ankle Outcome Score (FAOS). The authors concluded that fracture-dislocation occurred more frequently in patients who were older, female, and diabetic, and that the functional outcomes were generally poorer in comparison to ankle fractures without dislocation. Buyukkuscu et al. [16] compared the efficacy, functional outcomes, and complication frequency of splinting and external fixation in the initial treatment of ankle fracture-dislocations with poor soft tissue conditions. Patients were divided into two groups based on whether they were treated with a splint (*n* = 69) or an external fixator (*n* = 48). The authors reported that the frequency of loss of reduction (25% vs. 4%) and skin necrosis (22% vs. 6%) were significantly higher in the splint group, and the mean time period between injury and definitive surgery was significantly shorter in the external fixator group (11 ± 5 vs. 7 ± 4 days). However, in our cohort of patients, in which after reduction a temporary below the knee cast immobilization was applied, we observed, in line with other authors [17,18,19], satisfactory results at followup, without significant loss of reduction or skin complications. We applied an external fixator only in open fractures excluded from this study.

In the majority of cases of our series, we observed an involvement of the right side in younger patients in which the fracture occurred after a high energy trauma, while a fracture-dislocation secondary to a low-energy trauma occurred in six older women (66–79 years).

In eight cases, despite an excellent radiographic result (Van Djik 0), clinical evaluation showed some limitations (AOFAS < 90). 

Anatomic reduction seems an important factor to avoid late osteoarthritis; in our series, in all cases but one, in which good reduction was obtained, no radiographic signs of osteoarthritis were observed at followup.

In fracture-dislocations of the ankle, major wound complications are reported with a percentage between 3% and 44% [10,11,12]. We observed major wound complications only in two patients, but, in the present study, we excluded open fractures.

Early active and passive motion exercises seem to be an effective method both for allowing complete and quick recovery of the range of motion of the ankle and for reducing the risk of early degenerative joint disease [20].

This could mean that postoperative rehabilitation likely plays an important role in the final clinical outcome. 

There was a statistically significant association between OA and the quality of reduction. While it might not affect medium-term outcomes, any reduction that is not considered excellent, leads to some degree of arthritis, which might cause problems on long-term followups, over 10 years after surgery. Surprisingly, low-energy fracture-dislocations were associated with worse osteoarthritis scores. This can be, however, explained by the fact that patients with low-energy trauma were on average much older than those with high impact injuries; so, age is an important co-founder.

This study had some limitations. First, the absence of a control group of patients with ankle fractures without dislocation means this investigation cannot conclude how much of the clinical and radiological sequelae are due to the dislocation. A second limitation is the relatively small number of patients; so, some of the statistical analysis of the results may be underpowered. However, fracture-dislocations of the ankle are relatively uncommon, and, in the literature, little attention is given to the long-term results of these injuries. Our study reports clinical and radiographic results of a group of patients surgically treated for a fracture-dislocation of the ankle after a long-term followup. Moreover, in the present cohort we reported only cases with a complete dislocation of the talus, excluding partial dislocations that are sometimes reported in other studies.

## 5. Conclusions

Fracture-dislocations of the ankle are serious injuries of the bone and surrounding soft tissues of the ankle that are usually diagnosed after high-energy traumas, with some occurring after a low-energy trauma, especially in older women. At medium- to long-term followup, better radiographic results are generally related to a good reduction of the lesion; however, unsatisfactory functional results may be observed regardless of an anatomic reduction of the fracture-dislocation. In fact, in nine of our cases, the AOFAS score was <90 points, despite an excellent or good postoperative reduction.

## Figures and Tables

**Table 1:** Demographics, clinical, and radiological results of our cohort of patients.

Case	Sex—Age	Side	Energy	Type of Fracture	Weber Classif.	Quality of Reduction	Follow-Up Years	AOFAS	Radiograp. Results (Van Dijk)
1	M—20	R	High	Unimal.	B	Good	4.6	88	0
2	F—51	R	High	Unimal.	C	Excellent	6.8	92	0
3	F—39	R	High	Bimal.	B	Good	6.6	84	0
4	F—77	R	Low	Bimal.	C	Good	4.2	92	1
5	F—59	R	High	Trimal.	B	Good	4.6	96	1
6	M—45	R	High	Unimal.	A	Excellent	4.2	84	0
7	M—35	L	High	Bimal.	B	Excellent	5.3	88	0
8	F—68	R	Low	Bimal.	B	Excellent	5.8	90	1
9	F—26	R	High	Bimal.	B	Excellent	3.9	98	0
10	F—79	R	Low	Unimal.	C	Good	4.8	85	1
11	F—49	L	High	Bimal.	B	Good	5.2	75	1
12	M—59	R	High	Bimal.	C	Excellent	5.5	92	0
13	M—32	R	High	Bimal.	B	Excellent	5.3	82	0
14	M—24	R	High	Unimal.	B	Excellent	6.8	80	0
15	F—16	R	High	Unimal.	C	Excellent	3.7	96	0
16	F—45	R	High	Trimal.	B	Good	5.9	88	0
17	F—71	R	Low	Trimal.	B	Good	5.9	93	1
18	M—45	L	High	Unimal.	B	Excellent	3.8	85	0
19	F—24	R	High	Bimal.	C	Excellent	4.8	84	0
20	F—66	R	Low	Trimal.	B	Excellent	5.2	92	0
21	M—50	R	High	Unimal.	C	Excellent	6.1	94	0
22	F—45	L	High	Unimal.	B	Good	4.9	86	1
23	M—28	R	High	Bimal.	C	Good	5.6	76	1
24	F—74	R	Low	Trimal.	B	Good	6.2	88	1
25	F—28	R	High	Unimal.	C	Excellent	5.9	94	0
26	F—62	R	High	Bimal.	C	Good	4.4	85	1

## Data Availability

The data used and analyzed during in the current study are available from the first author on reasonable request.
